# Design, development, and validation of a non-backdrivable active ankle-foot orthosis for the TWIN lower-limb exoskeleton

**DOI:** 10.3389/frobt.2025.1647989

**Published:** 2025-08-18

**Authors:** Raffaele Giannattasio, Nicolò Boccardo, Riccardo Vaccaro, Heeral Bhatt, Stefano Maludrottu, Elena De Momi, Matteo Laffranchi

**Affiliations:** ^1^ Rehab Technologies Lab, Italian Institute of Technology, Genoa, Italy; ^2^ Department of Electronics, Information and Bioengineering, Politecnico di Milano, Milan, Italy; ^3^ Open University Affiliated Research Centre, Istituto Italiano di Tecnologia (ARC@IIT), Genova, Italy

**Keywords:** robotics, ankle-foot orthosis, rehabilitation, exoskeleton, minimum jerk, motion planning, multiple sclerosis, control

## Abstract

This study’s primary objective was to develop an Active Ankle-Foot Orthosis (AAFO) specifically designed for integration into lower-limb exoskeletons. An analysis of human ankle motion is conducted to inform the development process, guiding the creation of an AAFO that aligns with specifics extrapolated by real data. The AAFO incorporates an electric motor with a non-backdrivable transmission system, engineered to reduce distal mass, minimize power consumption, and enable high-precision position control. Capable of generating up to 50 Nm of peak torque, the AAFO is designed to provide support throughout the walking cycle, targeting pathological conditions such as foot drop and toe drag. Performance was first validated through benchtop experiments under unloaded conditions. The AAFO was then integrated into the TWIN lower-limb exoskeleton, employing an optimal trajectory planning method to generate compatible reference trajectories. These trajectories are designed to help the user maintain ground contact during the support phase while ensuring safe toe clearance and minimizing jerk during the swing phase. Finally, the AAFO’s performance was assessed in real-world application conditions, with four healthy participants walking with the TWIN lower limb exoskeleton. The results suggest that the proposed AAFO efficiently reduces toe clearance, ensures stable control, and maintains low power consumption, highlighting its suitability for clinical applications.

## 1 Introduction

According to the World Health Organization, spinal cord injury (SCI) (World Health Organization, and International Spinal Cord Society, 2013) and stroke (Owolabi et al., 2021) are the two leading causes of disability worldwide. Losing the ability to move the lower limbs negatively impacts quality of life and causes clinical complications such as muscle weakness and osteoporosis (World Health Organization, and International Spinal Cord Society, 2013). To prevent such complications and restore walking functionalities, rehabilitation plays a crucial role (Chung et al., 2019). In this context, lower-limb exoskeletons constitute a valuable robotic solution for rehabilitation, offering a range of benefits that allow them to effectively support conventional rehabilitation strategies (Mekki et al., 2018). Most lower-limb exoskeletons, including Ekso (Kolakowsky-Hayner, 2013), Indego (Farris et al., 2013), TWIN (Laffranchi et al., 2021), and Hal (Kawamoto et al., 2010), are designed to actively assist hip and knee flexion/extension motions using two actuation modules per leg. These modules typically rely on electric motors driving transmission systems engineered to achieve specific speed/torque joint performance that reflects human-like capabilities (Aliman et al., 2017). However, most of these devices are used in combination with passive ankle foot orthoses (PAFO), which employ passive elements, often featuring a spring with an adjustable damping mechanism, to modulate joint mobility (Esquenazi et al., 2012), allowing for passive dorsiflexion during single support while providing substantial resistance to plantarflexion during swing (Alam et al., 2014). Nevertheless, a PAFO cannot actively move the ankle during the walking cycle to enhance foot clearance, which is crucial for reducing the risk of stumbling in patients with neurodegenerative disorders like multiple sclerosis and stroke, muscle disorders, or nerve root injuries (NIH, 2024), who may experience difficulty lifting the front part of the foot, leading to the so-called “foot drop” problem (Jacquelin Perry, 1992). An active solution can mitigate these limitations by enabling dynamic regulation of the ankle joint position, improving foot clearance during the swing phase, and ensuring controlled ankle positioning during terminal swing. Recently, there has been an emphasis on developing exoskeletons with six degrees of freedom, allowing actuation of the hip, knee, and ankle joints in the sagittal plane. In this regard, the Mina Exoskeleton (Griffin et al., 2017; Mummolo et al., 2018) and Quix exoskeleton (Peterson et al., 2022) are outfitted with custom linear linkage actuators (LLA) for each of its six joints, utilizing frameless motors that drive a slider-crank linkage mechanism through a linear ball screw transmission. Similarly, the Exo H2 (Bortole et al., 2015) features a Brushless DC motor paired with a Harmonic Drive gearbox, which has a gear ratio of 160:1, to actuate each joint. However, the actuation mechanism used for the ankle joint in these systems typically mirrors that of the hip and knee joints (Bortole et al., 2015; Mummolo et al., 2018). This choice led to the development of a bulky actuation mechanism located near the ankle, leading to high joint inertia (Bortole et al., 2015). This increased inertia contributes to higher power consumption at the hip and knee joints during the swing phase of gait (Jin et al., 2017). Moreover, in such exoskeletons, the ankle joints are primarily designed to provide powered plantarflexion to assist push-off motion, generating torques of up to 120 Nm at high angular velocities (Peterson et al., 2022). This feature results in substantial energy expenditure per step, compromising the system’s overall energy efficiency and reducing the device’s operational endurance. Furthermore, although current exoskeletons with active ankle joints provide active dorsiflexion during the swing phase, they do not quantify how this ankle motion enhances the device’s ability to effectively increase toe clearance and prevent stumbling during level ground walking.

This paper presents the design and evaluation of a custom-made ankle actuation module intended for integration into the TWIN lower limb exoskeleton. The proposed ankle actuation module features a two-stage transmission system tailored to meet specific torque-speed requirements based on human biomechanics. Moreover, it was decided not to use the proposed ankle joint for active body propulsion, but rather to support the patient’s weight and ensure controlled foot clearance during the swing phase, thereby reducing the mechanical power needed to move the ankle during the walking cycle. In this regard, the proposed design ensures a non-backdrivable behavior, which is advantageous for minimizing power consumption during loaded conditions and achieving precise position control (Lenzi et al., 2019; Boccardo et al., 2023). The proposed design also enables the redistribution of the actuation module along the tibial link, reducing distal mass and allowing for a more compact configuration. Furthermore, it ensures modularity by supporting a reconfigurable link length. The proposed AAFO was subsequently integrated into the TWIN lower limb exoskeleton (Laffranchi et al., 2021) with an optimized trajectory planning method that aligns ankle motion with the pre-defined trajectories of the hip and knee joints. This approach was designed to mitigate the risk of stumbling, enhance patient comfort, and achieve a smooth motion at the ankle level (Giannattasio et al., 2024). The performance of the AAFO was initially assessed through test bench experiments and later evaluated in real-world scenarios through tests conducted with four healthy users. The experimental results demonstrate the device’s capability to accurately track human-like trajectories while maintaining precise position control and low power consumption during both walking and sit-to-stand/stand-to-sit tasks. Moreover, the use of the proposed active ankle during walking increases the minimum toe clearance compared to a passive configuration, demonstrating strong potential in reducing the likelihood of stumbling.

To provide a comprehensive overview of the research, Section 2 outlines the design methodology of the proposed AAFO, including system requirements, mechatronic design, and trajectory planning and control. Section 3 delineates the experimental methodology and presents the results from both test bench experiments and tests conducted on healthy users. Finally, Sections 4 and 5 discuss the potential impact of the proposed work and derive the conclusions.

## 2 Materials and methods

### 2.1 System requirements

The TWIN lower limb exoskeleton (Laffranchi et al., 2021) was developed with a user-centered approach at the Rehab Technologies Lab at the Italian Institute of Technology (IIT). TWIN features a modular design with four actuated joints at the hips and knees, powered by BMS-1712-A frameless motors (from Kollmorgen (Kollmorgen, 2024)) coupled with gearboxes of 80:1 and 50:1 ratios, respectively. Following a similar methodology, the subsequent sections will outline the system requirements for designing the AAFO intended for integration with the TWIN exoskeleton. This includes an explanation of the biological and technical requirements (Section 2.1.1), as well as considerations regarding power, safety, and motion aspects (Sections 2.1.2 and 2.1.3) that were incorporated throughout the development process.

#### 2.1.1 Human biomechanics and design requirements

The human ankle has variable kinetic and kinematic behaviors depending on the user’s walking speed (Grimmer et al., 2014). Additionally, the design of the actuation unit influences the amount of joint distal mass and can increase the energetic and metabolic cost of walking (Jin et al., 2017; Browning et al., 2007). These information are utilized to identify the following design features of the proposed ankle joint:

1. Kinematic: Range of Motions (RoMs), and speed must be comparable to those exhibited by humans during walking at a gait velocity that aligns with the maximum speed achievable by the TWIN exoskeleton. According to the literature on healthy walking (Grimmer et al., 2014), at a gait speed of 1.1m/s , the ankle joint exhibits a range of motion of 20° in dorsiflexion and 50° in plantarflexion and reaches a peak angular velocity of 4.3rad/s . Since the maximum gait velocity achievable by the TWIN exoskeleton is 0.33m/s (Giannattasio et al., 2024), these values are adopted as the conservative kinematic parameters for the development of the proposed active ankle joint.
2. Kinetic: During human walking, the kinetic demands of the ankle joint are highly dependent on the gait phase. During the support phase, the ankle supports the individual’s body weight (Torricelli et al., 2016). In this phase, the ankle velocity remains low, limiting the active power. During preswing, the ankle performs a powered push-off, generating a peak torque of 1.4Nm/kg and reaching a maximum joint velocity of 4.3rad/s (Grimmer et al., 2014) drastically increasing active mechanical power (Torricelli et al., 2016). Conversely, during the swing phase, the ankle joint attains a peak velocity of 2.62rad/s , while the torque remains relatively low (below 0.2Nm/kg ) due to the absence of load (Torricelli et al., 2016). To limit power consumption and reduce the maximum torque requirement, the proposed AAFO is designed to prevent stumbling during the unloaded swing phase rather than to generate propulsion. For this reason, the maximum torque requirement has been estimated to be below 50Nm , which corresponds to approximately 0.7Nm/kg for a 71kg individual, aligning with data presented in (Grimmer et al., 2014).
3. Modularity: The AAFO must be engineered to conform to the patient’s anthropometric dimensions, with the shank and foot lengths designed to be reconfigurable (Laffranchi et al., 2021).
4. Reduced distal mass: Most existing 6-DOF lower limb exoskeletons concentrate the actuation module near the ankle joint, resulting in a high distal mass of the AAFO (Bortole et al., 2015). An increased distal mass leads to greater joint inertia. As the inertia of the AAFO increases, power consumption at the hip and knee joints rises during the leg’s swing phase (Jin et al., 2017). Additionally, compensating for this increased inertia is more challenging for the exoskeleton, resulting in a higher metabolic cost for the user (Browning et al., 2007). To mitigate this, the AAFO inertia was reduced by redistributing the actuation module along the shank, shifting the center of mass closer to the tibia.

#### 2.1.2 Power consumption and safety requirements

As stated in (Louie et al., 2015), lower-limb rehabilitation sessions can last from 60 min to 2 h, in which the user can walk for about 100–120 m. Therefore, it is crucial to minimize motor power consumption to ensure sufficient battery life for extended use. Additionally, the actuator must be capable of withstanding peak loading forces that significantly exceed the maximum torques generated during walking, particularly in the event of a fall or improper use. To address these requirements, the ankle joint was designed to be non-backdrivable, enabling the system to resist static loads and sustain the patient’s weight without requiring motor torque, significantly reducing power consumption in static positions (Lenzi et al., 2019; 2017). Moreover, the joint remains static in response to sudden external load changes or power loss, ensuring more predictable control, improved user safety, and enhanced device durability (Boccardo et al., 2023).

#### 2.1.3 Motion control requirements

The human ankle plays distinct biomechanical roles throughout the gait cycle. During the support phase, it passively accommodates the kinematic constraints imposed by leg motion to maintain stability. In the preswing phase, it actively generates plantarflexion to contribute to forward propulsion. In contrast, during the swing phase, the ankle performs active dorsiflexion to improve toe clearance, reducing the risk of tripping and enabling smooth foot contact (Jacquelin Perry, 1992; Torricelli et al., 2016). As the proposed AAFO is not designed to provide powered push-off, the motion planning strategy focuses on replicating natural ankle behavior during the single support and swing phases, deliberately limiting plantarflexion during preswing. Moreover the trajectory planning method, introduced in (Giannattasio et al., 2024), incorporates the following criteria:

1. User comfort: The ankle trajectories are designed to promote comfort by ensuring smooth movements (Giannattasio et al., 2024). This is achieved through an optimization process that minimizes joint jerk, resulting in fluid and comfortable motion profiles.
2. Stumbling Prevention: Active dorsiflexion is provided during the swing phase to increase minimum toe clearance, significantly reducing the likelihood of stumbling (Rosenblatt et al., 2014; Lenzi et al., 2019).
3. Adaptability: The TWIN lower limb exoskeleton can execute various walking patterns by adjusting step height and step length. These parameters are manually adjusted according to the user’s specific clinical needs. These changes in walking patterns are reflected in the motion of the hip and knee joints, which adapt their behavior accordingly (Zuccatti et al., 2023). In response, the ankle joint trajectories are computed according to hip and knee joint movements, ensuring adaptability to gait variations (Giannattasio et al., 2024).

### 2.2 AAFO mechatronics design

This section outlines the mechatronic design of the proposed AAFO, developed to meet the design requirements specified in Section 2.1.

#### 2.2.1 Mechanical design

**FIGURE 1:**
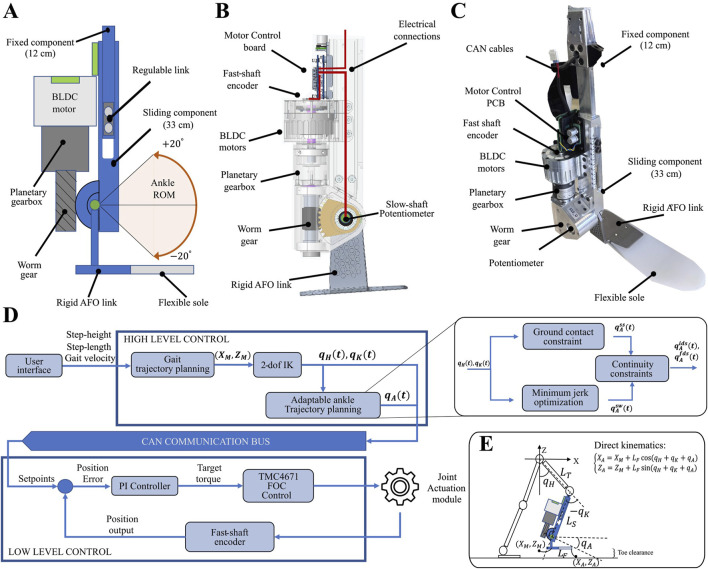
Upper part: **(A)** Simplified diagram of mechanical design, **(B)** Placement of the mechanical components, **(C)** The real AFO device. The fixed component of the AAFO is attached to the knee joint. The sliding component, which houses the proposed actuation system, can be moved along a linear guide within the fixed section to modulate the shank length. Lower part: **(D)** Overall System Architecture: The user interacts with the high-level control system to define gait parameters. The high-level control system performs adjustable gait trajectory planning based on user input. Hip and knee reference trajectories are computed using inverse kinematics. These computed hip and knee trajectories are subsequently used to derive adaptable reference trajectories for the ankle joint. The reference signals are sent to the low-level controller via the CAN bus. The low-level controller performs a PI position control to command the actuators. **(E)** Simplified kinematic model of the Twin lower-limb exoskeleton.

To enhance modularity while distributing the actuation module along the tibia, the tibial link consists of two components: a fixed component (12 cm in length) and a sliding component (33 cm in length). The actuation system is securely mounted on the sliding component, which moves along an integrated linear guide within the fixed section (see Figure 1A). This two-part design allows the actuation unit to be positioned along the link, reducing the distal mass, while also providing modularity to accommodate users with tibia lengths ranging from 33 cm to 45 cm, accurately covering 81% of the population’s average tibia length (Aitken, 2021). As depicted in Figure 1A, the actuation system of the proposed AAFO is designed with a single axis of rotation, to allow for plantarflexion and dorsiflexion movements in the sagittal plane. The RoM is configured to accommodate ± 20 degrees of plantarflexion and dorsiflexion, aligning with the specified kinematic design requirements. The overall actuation system consists of an ILM50x14 frameless motor (from TQ-RoboDrive (tq group, 2024)) driving a two-stage transmission. The first stage is a planetary gearbox (GPL042, from Gysin (gysin, 2024)) comprising one sun gear, a ring gear, and four planet gears, providing a reduction ratio of [4:1]. This configuration reduces the motor’s Revolutions Per Minute (RPM) while maintaining high efficiency ( η≃ 0.9), ensuring sufficient torque to drive the subsequent gear stage. The second stage consists of a worm gear (from Mädler (maedler, 2024) with a [35:1] ratio, selected to achieve the required speed/torque performance specified in Section 2.1.1 while minimizing the gear’s overall volume. Additionally, the worm gear’s low efficiency ( η≃ 0.43) ensures non-backdrivability, preventing reverse motion of the mechanism. This feature, combined with an appropriate frame design, allows the ankle to support the patient’s full weight in a stationary position without battery consumption (self-locking condition). As a result, the user can rely on the ankle mechanism during high-load activities, such as standing or sitting, enhancing stability and reducing physical effort. As shown in Figure 1B, the incorporation of the worm gear facilitates the reversal of the motion axes, enabling the redistribution of the entire actuation unit along the tibia. This configuration leads to a more compact AAFO design with a reduced distal mass. To effectively transfer motion to the foot, the terminal section of the AAFO consists of two components (Figure 1C): a rigid part directly connected to the slow shaft and a polypropylene sole, designed to emulate the stiffness of the human foot. The sole can be attached or detached from the AAFO, allowing for a modular configuration with five size options to accommodate different foot lengths, ensuring a user-centered design.

#### 2.2.2 Electrical design

The onboard electronics include a microcontroller (TMC1294KCPDT (TexasInstruments, 2024)) that communicates with the high-level control system via CAN bus, receiving reference signals at a frequency of 500 Hz. The AAFO is equipped with reflective optical encoders (IC-PR2656, from icHaus) that provide 4096 cycles per revolution. These encoders, mounted on the motor’s fast shaft, transmit motor position data to the motor controller. At the controller, the data is multiplied by the transmission reduction ratio to obtain precise position feedback. Joint movement is regulated by a proportional-integral (PI) position controller, implemented using a single fully integrated servo controller (TMC4671 (AnalogDevices, 2024)), which receives reference signals and executes an internal Field-Oriented Control (FOC) to command the actuator (see Figure 1D). Additionally, a potentiometer (metallux, 2024) is directly placed on the slow shaft, providing additional position feedback to the TMC4671, ensuring accurate FOC performance.

### 2.3 Trajectory planning and control

As illustrated in Figure 1D, the software architecture of the TWIN exoskeleton is divided into two layers. The high-level control layer generates joint trajectories based on user preferences, which are manually defined through a user interface. It provides references via the CAN bus to the second layer, the low-level control. In the low-level control, a PI position controller is implemented for each joint, which generates reference signals for the TMC4671 module that performs an inner FOC loop to command the actuators. The hip and knee trajectories are obtained from the walking gait trajectory using a 2-degree-of-freedom (2-DOF) inverse kinematics algorithm computed in real time. As stated in (Zuccatti et al., 2023), the gait trajectory of TWIN is computed by subdividing the human gait cycle into four distinct phases: initial double support (IDS), single support (SS), final double support (FDS), and swing (SW). Where FDS and IDS correspond to the preswing and load response phases Jacquelin Perry (1992). Each phase is modeled using a two-dimensional n-degree Bézier curve, mathematically defined by a set of Cartesian control points. A subset of these control points is selected to ensure ${\text{C}}^{3}$ continuity between the successive gait phases. The remaining control points are adjusted to modify the gait pattern, allowing for variations in step height (SH) and step length (SL), as specified by user preferences entered through a graphical user interface (GUI) that interacts with the high-level control. This approach enables personalized modulation of gait trajectories, tailoring hip and knee joint motions to the specific user needs. The resulting hip and knee trajectories are utilized in the proposed ankle trajectory planning method to generate ankle reference trajectories that adapt to variations in the walking pattern. This section details how the desired motion behavior of the ankle joint is achieved and incorporated into the high-level control framework of the TWIN exoskeleton. Subsequently, Section 2.3.1, based on the methodology introduced in (Giannattasio et al., 2024), details the optimal trajectory generation algorithm for the ankle joint and its integration within TWIN’s high-level control architecture.

#### 2.3.1 Optimal ankle trajectories

Following the motion requirements outlined in Section 2.1.3, the ankle trajectory must be designed to maximize user comfort while ensuring sufficient toe clearance to prevent stumbling and promote adaptability to changes in the desired walking pattern. To satisfy these objectives, the ankle trajectory is generated based on the precomputed hip and knee motions (Figure 1D), following the methodology presented in (Giannattasio et al., 2024). To accurately represent the distinct biomechanical behavior of the ankle throughout the gait cycle, the trajectory is segmented into four polynomial functions, each corresponding to a specific gait phase and defined as follows:

• Single Support Polynomial $\left(q_{A}^{ss}\left(t\right)\right)$ : This polynomial is designed to satisfy the ground contact constraints imposed on the ankle by the hip and knee angles during the single support phase. Referring to Figure 1E, the constraint is derived by imposing $z_{A}\left(t\right)=z_{M}\left(t\right)$ . Where $z_{A}\left(t\right)$ denotes the vertical position of the foot tip, while $z_{M}\left(t\right)$ denotes the vertical position of the ankle joint. By applying direct kinematics, the required ankle angle to maintain ground contact, $q_{A}\left(t\right)$ , is determined as a function of the hip and knee angles. Using this relationship, the hip and knee angles are utilized to generate a set of waypoints that the ankle joint must satisfy to maintain full ground contact over the entire single support phase. The ankle trajectory is subsequently obtained by solving a least-squares minimization problem (Giannattasio et al., 2024), resulting in a polynomial that optimally fits the established set of waypoints.

• Swing Polynomial $\left(q_{A}^{sw}\left(t\right)\right)$ : This polynomial is computed by solving a constrained optimization problem. A jerk minimization strategy (Piazzi and Visioli, 2000; Amirabdollahian et al., 2002) is employed to achieve smooth joint motions. Initial and final conditions on position, velocity, and acceleration are imposed to ensure a safe transition at the start of the swing and a smooth landing on the ground. To further enhance safety, linear and nonlinear constraints are incorporated into the optimization problem. These constraints promote ankle dorsiflexion during initial swing, enforce adherence to the mechanical range of motion, and confine the trajectory within a safety region designed to represent the condition necessary to avoid stumbling. Refer to (Giannattasio et al., 2024) for further implementation details.

• Initial and Final Double Support Polynomials $\left(q_{A}^{\mathrm{ids}}\left(t\right),q_{A}^{\mathrm{fds}}\left(t\right)\right)$ : These polynomials are computed to ensure smooth transitions, imposing continuity constraints in position, velocity, and jerk between the single support and swing polynomials. This approach ensures smooth and continuous trajectories while also significantly limiting the maximum plantarflexion during the final double support phase, thereby reducing the powered push-off motion (Giannattasio et al., 2024).

## 3 Experiments and results

To validate and characterize the proposed AAFO, a set of simulations was performed, followed by benchtop experiments and tests with healthy participants. The simulations aimed to assess the behavior of the proposed trajectory planning algorithm, while the benchtop experiments evaluated the ankle joint’s performance in terms of position control bandwidth. The tests with healthy participants focused on assessing the ankle joint’s behavior under real-world application conditions and verifying its alignment with the design requirements outlined in Section 2.1.

### 3.1 Simulations

A series of simulations were conducted in MATLAB (version 2021b) to evaluate the effectiveness of the proposed ankle trajectories in meeting the motion-control requirements outlined in Section 2.1.3. Initially, the gait pattern generator described in (Zuccatti et al., 2023) was used to generate reference gait trajectories. Subsequently, a 2-DOF inverse kinematics approach was applied to derive the corresponding hip and knee joint angles. These angles were then used to compute the ankle trajectories following the proposed approach from (Giannattasio et al., 2024). This procedure was repeated with different combinations of step length and step height parameters, summarized in Table 1. Simulations were divided into two tests:

1. Test 1: The ankle range of motion in human walking increases with gait speed (Grimmer et al., 2014), characterized by a greater dorsiflexion during the single support phase while the foot maintains full ground contact. This test aims to evaluate the integration of this biomechanical feature within the proposed ankle trajectory planning method. To this end, simulations were performed with a fixed step height of 6 cm and systematically varied step length (between 30 cm and 40 cm), as increases in step length directly enhance forward progression and consequently gait speed.
2. Test 2: Walking with a reduced step height directly decreases minimum toe clearance, thereby increasing the risk of stumbling (Rosenblatt et al., 2014). This test was designed to demonstrate the effectiveness of the proposed trajectory in enhancing minimum toe clearance across varying step height conditions. To evaluate this, simulations were conducted with a fixed step length of 30 cm while systematically varying the step height between 2 cm and 10 cm. Referring to Figure 1E, toe clearance was determined as the vertical difference between the foot tip position in swing (computed via direct kinematics) and the ground. Toe clearance was analyzed under two conditions: For the first condition (AAFO condition), toe clearance was computed using the proposed ankle trajectories. For the second condition (PAFO condition), the ankle angle was assumed to remain fixed at the dorsiflexed position reached at the end of the support phase. This assumption aligns with the behavior of commercial PAFOs, which employ a stiffness-damping mechanism to allow passive dorsiflexion during single support while providing substantial resistance to plantarflexion during swing, preventing inertia-driven deviations from the dorsiflexed position (Alam et al., 2014).

**TABLE 1:** Description of the conditions simulated during Test one and Test 2.

Test	Step length	Step height	$L_{T}$	$L_{S}$	$L_{F}$
Test 1	30,32,34,36,38,40 (cm)	6 cm	38 cm	46 cm	18 cm
Test 2	40 cm	2,4,6,8,10 (cm)	38 cm	46 cm	18 cm

For both tests, a value of $L_{T}=38\;cm$ , $L_{S}=46\;cm$ , and $L_{F}=18\;cm$ were used to simulate the system kinematics.

**FIGURE 2:**
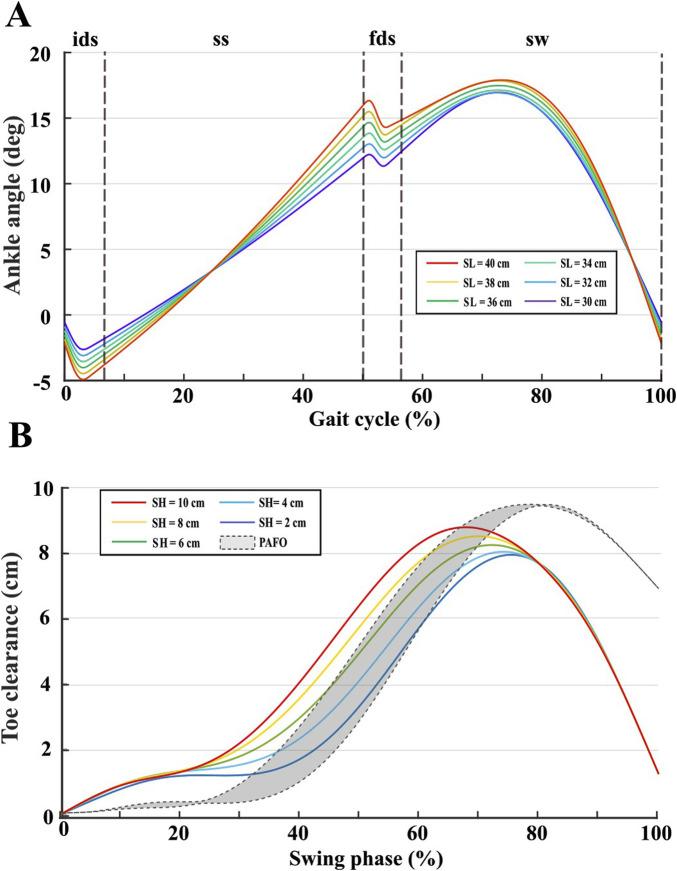
**(A)** Ankle trajectories over the gait cycle percentage, for a fixed step height (6 cm) and variable step length (30 cm–40 cm). **(B)** Toe clearance over the swing phase percentage, for a fixed step length (40 cm) and variable step height (2 cm–10 cm). The grey shaded area represents the toe clearance under the PAFO condition across all tested step heights.

Figure 2A presents the ankle joint trajectories during the gait cycle for the simulations conducted in Test 1. The results demonstrate the ankle’s ability to adapt its trajectory in response to variations in step length, with longer steps resulting in a greater range of motion, which is essential for meeting the ground contact constraints imposed by the hip and knee joints during the single-support phase. Figure 2B illustrates the toe clearance during the swing phase for the simulations conducted in Test 2. The results emphasize the advantages of active ankle motion. During the initial swing phase, where the risk of stumbling is highest, the AAFO simulation achieves greater minimum toe clearance than the PAFO simulations, effectively reducing the likelihood of tripping and enhancing safety. In the later swing phase, controlled plantarflexion gradually decreases toe clearance, promoting a smoother landing and seamless transition into the support phase.

### 3.2 Bandwidth analysis

**FIGURE 3:**
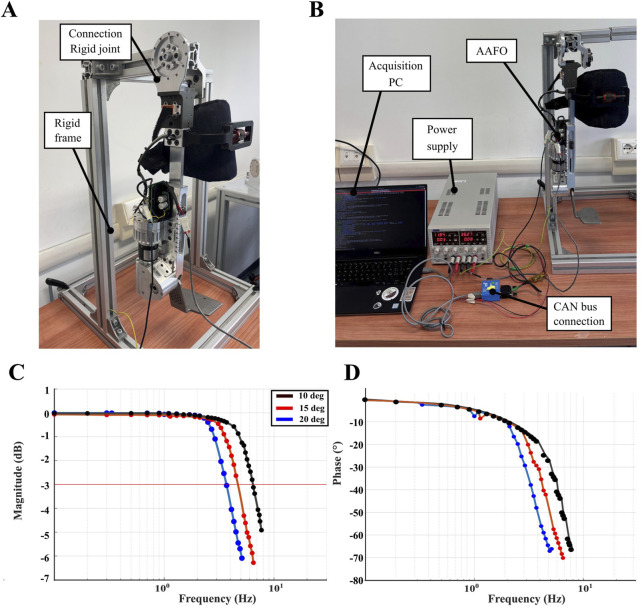
Lateral **(A)**, and frontal view **(B)** of the testbench used to characterize the ankle joint behavior. Magnitude **(C)** and phase **(D)** Bode diagram representing the frequency response of the position control loop for the ankle joint at the three different input conditions.

A closed-loop position bandwidth test was performed to assess the ability of the proposed actuator to track reference signals at different speeds. This test was designed to determine the maximum frequency the ankle actuator can operate while maintaining accurate tracking of the reference signal. For this test, the AAFO was securely mounted on a benchtop and controlled via a CAN bus using a laptop (see Figures 3A,B). To characterize the bandwidth, a sinusoidal position reference signal of constant amplitude was transmitted to the position controller at a frequency of 500 Hz. The reference sinusoid frequency was initially set to 0.1 Hz and gradually increased until the test was terminated due to excessive shaking. Given that the range of maximum dorsiflexion achieved by the human ankle during gait is typically between 10 and 20° (Jacquelin Perry, 1992), the test was conducted using three different reference amplitudes: 10, 15, and 20°. Figure 3C,D present the Bode plots for closed-loop position bandwidth tests, illustrating the cutoff frequencies for each of the three test scenarios. The cutoff frequencies are identified as the points where the magnitude of the system response intersects the red −3 dB attenuation line. The identified cutoff frequencies are 6.4 Hz, 4.7 Hz, and 3.7 Hz for input sinusoidal references of 10°, 15°, and 20°, respectively. It is important to highlight that even under the most challenging conditions (reference amplitude of 20°), a cutoff frequency of 3.7 Hz was achieved. Considering that frequency analysis of human gait indicates that the highest frequency components fall within the range of 1.3–2.3 Hz, achieved respectively walking at a gait speed of 0,55 and 2.33 m/s (Louie et al., 2015), the actuator is expected to be fully capable of accurately tracking human-like joint trajectories.

### 3.3 Standing, sitting, and walking tests

**FIGURE 4:**
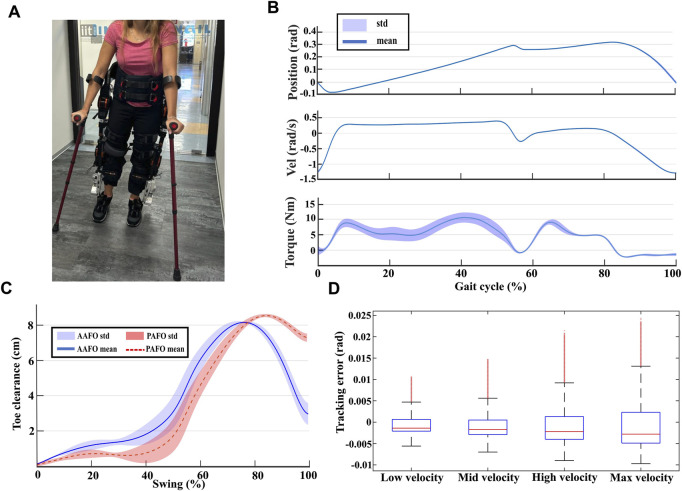
**(A)** Snapshot of an experiment. **(B)** Mean plus standard deviation of ankle joint positions, velocities, and torques for user two walking at maximum speed. **(C)** Average toe clearance computed for all the users for active ankle condition (in blue), and for passive ankle condition (in red). **(D)** Box plot of the position tracking error at the ankle joint averaged between all users for the four tested gait speeds.

A study was conducted with four healthy participants who had prior experience walking with the TWIN lower-limb exoskeleton. During all the tests, participants use crutches to maintain balance in the frontal plane (see Figure 4A). To ensure broader generalization, the participants exhibited diverse characteristics in terms of weight and anthropometric parameters. Due to the modular design of the TWIN exoskeleton, the dimensions of its links were adjusted before each experiment to align with the individual user’s limb measurements. Additionally, the AAFO sole was replaced to match the user’s foot length. Table 2 summarizes the users’ body weight alongside the corresponding exoskeleton kinematic parameters $\left(L_{T},L_{S},L_{F}\right)$ . Where $L_{T}$ and $L_{S}$ denote the distances from the hip to the knee joint and from the knee to the ankle joint, respectively, matching the user’s thigh and shank lengths. Meanwhile, $L_{F}$ represents the portion of the foot from the ankle joint to the foot tip, which is about 7 cm shorter than the user’s foot length. The experimental protocol was approved by comitato etico regione Liguria (protocol code: N. CET - Liguria: 297/2024 - DB id 14017). The studies were conducted according to the local legislation and institutional requirements. The participants provided their written informed consent to participate in this study.

**TABLE 2:** Users’ weight and kinematics characteristics.

User	Weight	$L_{T}$	$L_{S}$	$L_{F}$
User 1	58 kg	39 cm	44 cm	19 cm
User 2	62 kg	40 cm	46 cm	20 cm
User 3	54 kg	38 cm	43.9 cm	19 cm
User 4	75 kg	41 cm	47.1 cm	21 cm

#### 3.3.1 Experimental setup

The experimental protocol comprised two primary test categories: 10-m walking tests (10MWT) on level ground and sit-to-stand/stand-to-sit tests. During the 10MWT, participants were instructed to walk along a 10-m straight path under varying gait conditions, summarized in Table 3. The tests were performed at four different gait speeds, ranging from the lowest one (0.15m/s) to the maximum achievable by the TWIN exoskeleton (0.33m/s) . These speeds, defined as the ratio between the step length and the walking period, were selected to align with the average speed of 0.26m/s , typically achieved by commercial exoskeletons during rehabilitation for individuals with SCI (Louie et al., 2015). To assess the ankle joint’s capability to prevent stumbling, all tests were performed with a step height of 10 cm, which was then progressively reduced to 2 cm. During the test, the method described in (Zuccatti et al., 2023) was used to compute reference trajectories for the hip and knee joints, while the ankle trajectories were computed following the method introduced in (Giannattasio et al., 2024).

**TABLE 3:** Experimental testing procedure.

Test name	Test type	Step length	Step height	Gait period	Gait speed
Low velocity walk	10 MWT	30 cm	2,4,6,8,10 (cm)	2 s	0.15 m/s
Mid velocity walk	10 MWT	35 cm	2,4,6,8,10 (cm)	1.7 s	0.205 m/s
High velocity walk	10 MWT	40 cm	2,4,6,8,10 (cm)	1.4 s	0.285 m/s
Max velocity walk	10 MWT	40 cm	2,4,6,8,10 (cm)	1.2 s	0.33 m/s
Sitting/Standing	5 repetition test	-	-	-	-

The sit-to-stand/stand-to-sit tests consist of five repetitions of sit-to-stand/stand-to-sit motions. During the sit-to-stand test, participants were instructed to lean forward while the exoskeleton executed hip and knee extension to pass into the standing position. Conversely, during the stand-to-sit test, participants were asked to lean backward while the exoskeleton executed hip and knee flexion to return to the sitting position. Throughout these tests, the ankle joint was held at a fixed reference position of zero degrees. This configuration, combined with the non-backdrivable design of the AAFO, enabled participants to rely on ankle support without affecting the power required to compensate for external loads, ensuring a stable execution of the movement.

#### 3.3.2 Performance metrics

Joint positions, velocities, currents, and electrical powers were collected during the tests to evaluate the AAFO behavior in matching the system requirements outlined in Section 2.1. The slow shaft velocity was derived by dividing the fast shaft encoder measurement by the total gear ratio of the mechanism. Joint positions were used to compute toe clearance using direct kinematics. To compute toe clearance under the PAFO condition, the ankle angle was held fixed at the dorsiflexed position attained at the end of the support phase. The motor current, $i_{\mathrm{motor}}$ , was used to estimate the slow shaft torque based on Equation 1.

$$\tau =i_{\mathrm{motor}}*K_{t}*G_{\mathrm{plan}}*\eta_{\mathrm{plan}}*G_{\mathrm{worm}}*\eta_{\mathrm{worm}}$$ (1)

Where $K_{t}$ represents the motor torque constant, while $G_{\mathrm{plan}}$ , $\eta_{\mathrm{plan}}$ , $G_{\mathrm{worm}}$ , and $\eta_{\mathrm{worm}}$ correspond to the gear ratio and efficiency of the planetary and worm gears, respectively. Toe clearance was computed using direct kinematics according to the joint positions (Figure 1E). To simulate PAFO behavior, the ankle joint during swing was supposed to remain fixed at the dorsiflexed position acquired at the end of the support phase.

All collected data were analyzed during post-processing. Specifically, the data from each experiment was segmented into individual strides. To achieve a robust statistical representation, the stride data for each exercise across all participants were averaged, providing a comprehensive depiction of the kinetic and kinematic behavior of the ankle joint. The averaged peak ankle velocities and peak ankle torques were analyzed to assess whether the actuation system operates under conditions compatible with the design specifications outlined in Section 2.1.1. The position tracking error at the ankle joint was measured to assess the performance of the proposed control strategy. Toe clearance values were averaged across all participants. Minimum toe clearance achieved with the proposed AAFO during the swing phase was compared to that of the PAFO to assess the AAFO’s effectiveness in reducing the likelihood of stumbling. Finally, the electrical power consumed at the ankle joint was compared to that of the hip and knee joints to assess the impact of the proposed AAFO on the overall energy demand of the system, and consequently, its effect on battery life during ambulation with the TWIN exoskeleton.

#### 3.3.3 Kinematic and kinetic behavior

Figure 4B presents the mean and standard deviation of ankle position, velocity, and torque at the slow shaft throughout the gait cycle, for user two walking at the maximum speed achievable by the exoskeleton (0.33m/s) . This scenario was selected to represent the worst condition since, during this test, the ankle joint experienced the highest mechanical power. From Figure 4B, it can be observed that the ankle joint provides higher torque during the support phase ( 10% - 50% of the gait cycle) as it must sustain the user’s weight. In contrast, during the swing phase ( 60% - 100% of the gait cycle), the torque remains relatively low due to the foot’s low inertia. Moreover, the standard deviation of torque data remains consistently low throughout the swing phase. Since torque deviations are linked to uncomfortable foot accelerations, the observed low variance represents a desirable feature for enhancing motion comfort. Angular velocity remains low during the support phase and reaches its maximum at the end of the swing phase.

Table 4 shows the average peak ankle velocities and torques, normalized by body weight, recorded across all participants at different gait speeds. Torque values were generally consistent among users. The highest torque was observed in User 4, who had the highest body weight (75Kg) , resulting in a peak torque value of 13.5±2.22Nm . In contrast, the maximum peak angular velocity, $-1.37\pm 0.024\;\frac{rad}{s}$ , was recorded for user 3, who had the shortest thigh and shank lengths, necessitating higher joint velocities to complete the gait cycle within the specified walking period. These values remain below the torque and velocity requirements outlined in Section 2.1.1, indicating that the proposed AAFO mechanism is appropriately dimensioned. It effectively accommodates the tested users and provides a sufficient margin to support individuals with greater body mass and limb lengths.

**TABLE 4:** Average peak velocity and peak torque at ankle level for all the users walking at different gait speeds.

User	Low velocity	Mid velocity	High velocity	Max velocity
User1 peak velocity $\frac{rad}{s}$	−0.59±0.034	−0.82±0.01	−1.13±0.03	−1.35±0.04
User1 peak torque $\frac{Nm}{Kg}$	0.12 ± 0.003	0.16 ± 0.003	0.2 ± 0.003	0.17 ± 0.005
User2 peak velocity $\frac{rad}{s}$	−0.55±0.01	−0.8±0.01	−1.12±0.04	−1.31±0.02
User2 peak torque $\frac{Nm}{Kg}$	0.11±0.024	0.12±0.04	0.15±0.04	0.19±0.039
User3 peak velocity $\frac{rad}{s}$	−0.6±0.01	−1.02±0.02	−1.17±0.035	−1.37±0.024
User3 peak torque $\frac{Nm}{Kg}$	0.17±0.04	0.21±0.03	0.18±0.04	0.17±0.03
User4 peak velocity $\frac{rad}{s}$	−0.41±0.01	−0.57±0.02	−0.78±0.02	−0.9±0.02
User4 peak torque $\frac{Nm}{Kg}$	0.084±0.02	0.16±0.04	0.17±0.04	0.18±0.03

#### 3.3.4 Position control behavior and safety considerations

Figure 4D presents the position control tracking error averaged between all users walking at various gait speeds, depicted as a boxplot. The figure reveals that the magnitude of the control error increases with gait speed, and consequently with gait frequency, as anticipated from the frequency analysis shown in Figure 2. Additionally, the error amplitude reaches a maximum mean value of −0.005rad , with an outlier value of 0.025rad in the worst condition. This error is considered negligible and does not significantly affect the system’s performance.

Figure 4C shows the mean toe clearance as a function of swing phase percentage, obtained by averaging the toe clearance values of all participants and walking conditions. The blue line represents the average toe clearance for the AAFO condition, while the red dotted line corresponds to the PAFO condition. Shaded regions indicate the standard deviations. The results indicate that minimum toe clearance in the AAFO condition is significantly higher than in the PAFO condition, with a minimum toe clearance of 1.3 cm for AAFO compared to 0.3 cm for PAFO. These findings highlight the effectiveness of the proposed AAFO in enhancing toe clearance and reducing the risk of stumbling, aligning with the motion requirements outlined in Section 2.1.3.

#### 3.3.5 Power consumption considerations

**FIGURE 5:**
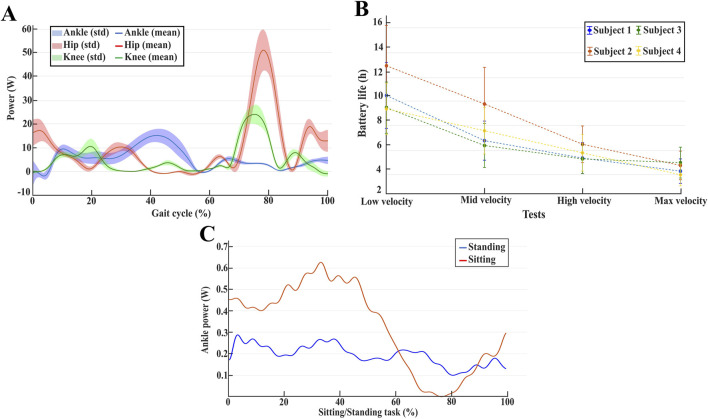
**(A)** Mean and standard deviation of power consumption for hip, knee, and ankle joint for user two walking at maximum gait speed. **(B)** The average estimated battery life for all the users at different gait speeds. **(C)** Ankle power consumption averaged between all users during the sit-to-stand and stand-to-sit tasks.

Figure 5A compares the mean electrical power consumption of the ankle joint during the gait cycle with that of the hip and knee joints. The comparison indicates that the power required to actuate the ankle joint during a step is lower than that needed for the hip and knee joints, and therefore has a minimal impact on the overall energetic cost of the system.

By integrating the curves shown in Figure 5A over the stride period, the average electrical energies were calculated. Moreover, the total average energy used during one gait cycle was determined using Equation 2.

$$E_{\mathrm{mean}}=2*\int_{0}^{T_{\mathrm{stride}}}p_{A}\left(t\right)+p_{H}\left(t\right)+p_{K}\left(t\right)dt$$ (2)

Where $p_{A}\left(t\right),p_{H}\left(t\right),p_{K}\left(t\right)$ represent the averaged power consumption for each joint, while $T_{\mathrm{stride}}$ represents the stride period. Assuming that each leg experiences the same averaged power consumption during the gait cycle, $E_{\mathrm{mean}}$ represents the total estimated energy consumed by the exoskeleton at each stride. It contains the average energy contribution of each joint for both legs. Comparing this energy with the maximum amount of energy that the exoskeleton battery pack can give $\left(E_{\mathrm{battery}}\right)$ , it is possible to estimate the maximum duration of the battery while performing a specific exercise with the TWIN exoskeleton equipped with the ankle joint using Equation 3:

$$BatteryLife=\frac{E_{\mathrm{battery}}}{E_{\mathrm{mean}}}*T_{\mathrm{stride}}$$ (3)

Figure 5B shows the averaged battery life computed for all the users at different gait speeds. In terms of battery life, the most demanding condition occurs when walking at maximum gait speed. However, the battery life still exceeds 2 hours of continuous use. Since rehabilitation sessions typically last less than 2 hours (Louie et al., 2015), this condition is considered robust for clinical applications.

Figure 5C presents the averaged power consumption at the ankle joint during sit-to-stand and stand-to-sit tasks. To ensure consistency across all participants, the duration of these motions was standardized. During these tests, the users rely on the ankle joint to tilt their body to complete the tasks. As a result, these tasks require the ankle joint to sustain high loads while remaining static (fixed at zero degrees). As seen in Figure 5C, the averaged power consumption at the ankle joint remains low throughout both sit-to-stand and stand-to-sit transitions. This outcome reflects the non-backdrivable nature of the joint, which allows the mechanism to remain unaffected by external loads, thereby maximizing energy efficiency. As a result, low power is required to maintain a static position, demonstrating the joint’s ability to support the user’s weight without additional energy expenditure.

The presented results demonstrate that integrating the proposed AAFO into the TWIN exoskeleton does not compromise the device’s usability in terms of energy consumption during both level ground walking and sit-to-stand/stand-to-sit tasks, aligning with the power consumption requirements discussed in Section 2.1.2.

## 4 Discussion

The experimental results indicate that the proposed AAFO performs effectively when tested on healthy users at gait speeds comparable to those of commercial exoskeletons (0.26m/s) , successfully meeting the design criteria outlined in Section 2.1. The proposed trajectory planning method effectively synchronizes ankle motion with hip and knee movements, allowing adaptability to variations in step length and step height. The AAFO also demonstrated precise control performance, tracking the reference signal with a maximum error of 0.03rad . A maximum speed of 1.37±0.024rad/s and a peak torque of 13.5±2.22Nm were achieved during testing. These torque-speed characteristics respect the kinetic and kinematic design requirements introduced in Section 2.1.1, and are significantly lower than those observed in other state-of-the-art exoskeletons with active ankle joints, which provide torques up to 120Nm during powered plantarflexion (Peterson et al., 2022). The low torque variance observed during the swing phase highlights the ability of the minimum jerk trajectory planning to enhance smooth motions and user comfort. Additionally, the proposed AAFO requires relatively low electrical power to move during the gait cycle, and its integration does not negatively impact the exoskeleton’s efficiency in terms of battery life. The estimated average battery life indicates that the exoskeleton, equipped with the proposed ankle joint, can operate continuously for over 2 hours, demonstrating its suitability for clinical applications. This energy-efficient design comes at the cost of not providing active push-off assistance, which could hinder the user during weight shifting. However, the TWIN gait trajectories are specifically designed to support weight shifting, partially compensating for the absence of push-off (Zuccatti et al., 2023). Moreover, users rely on crutches to facilitate weight transfer between steps. Therefore, prioritizing energy efficiency over powered propulsion is regarded as a reasonable and effective design compromise for the proposed AAFO.

Joint angle positions collected during walking trials were used to compute toe clearance for both the AAFO and PAFO conditions using direct kinematics. The resulting toe clearance values are relative to the exoskeleton. However, because the exoskeleton’s link lengths are tailored to each participant’s anthropometric measurements and the AAFO sole is replaced to match the user’s foot length, any discrepancy between the exoskeletons and the user’s toe clearance is effectively negligible. Furthermore, the exoskeleton is equipped with a very thin sole, and participants wore conventional shoes during the trials, further minimizing potential deviations. Toe clearance values were averaged across all participants, with the mean and standard deviation presented in Figure 4C. Across all tests, the minimum toe clearance observed was 1.3 cm for the AAFO condition and 0.3 cm for the PAFO condition. According to data from (Rosenblatt et al., 2014), a 15 mm increase in minimum toe clearance during level-ground walking could reduce the probability of contacting a 5 mm obstacle from one in 150 steps to one in 4000 steps. In the tested scenarios, the proposed AAFO achieved a 10 mm increase in minimum toe clearance compared to a passive solution, indicating a strong potential to significantly reduce fall risk during level-ground walking, regardless of the user’s compensatory mechanisms for stumbling prevention. Moreover, the minimum toe clearance values observed under AAFO conditions are consistent with those typically seen in human walking (Schulz, 2011). This highlights the potential of the proposed AAFO to replicate human-like gait characteristics.

It is important to note that the positive findings presented in this study are based on tests done for a short period under laboratory settings involving only four healthy participants. Consequently, the same outcomes cannot be guaranteed in clinical settings. SCI patients often experience conditions such as spasticity and other neuromuscular impairments. These conditions may introduce disturbance torques that could degrade control performance and increase power consumption. However, the non-backdrivable joint design, in combination with a flexible sole, helps mitigate the transmission of such disturbance torques. Furthermore, the experimental results reported in this work show that the active torque and power delivered by the AAFO during trials with healthy participants remain below the device’s maximum capability. Thus, any potential degradation in performance observed during testing with actual patients is expected to remain within manageable limits.

The current testing methodology also reflects the general limitations of a lower limb exoskeleton. For example, the maximum gait speed evaluated is constrained by the actuator’s capabilities, and it is still low compared to the average gait speed achieved in human walking (Grimmer et al., 2014). In addition, gait parameters are manually selected, suggesting that the device lacks the ability to integrate the user’s intent into the control system. Furthermore, the TWIN exoskeleton has only six DOF, which constrains user movement within the sagittal plane.

In conclusion, a more extensive clinical validation involving individuals with SCI is necessary to fully assess the performance of the proposed AAFO in clinical settings. To this end, future studies will focus on evaluating the AAFO in clinical environments under long-term experimentation, with particular attention to user experience and potential performance degradation. The insights gained from these trials will provide essential feedback to guide further improvements in both hardware design and control strategies. Future research will also focus on optimizing overall exoskeleton motions to enable faster gait velocities and developing more user-centered control strategies that can adjust gait parameters online based on user intention. Moreover, since the proposed AAFO is actuated along a single degree of freedom, future research endeavors will be needed to investigate the trade-offs involved in incorporating additional degrees of freedom into the joint design.

## 5 Conclusion

This paper presented the design, development, and validation of a non-backdrivable AAFO intended for integration into the TWIN lower limb exoskeleton. The design process was guided by a comprehensive set of requirements, including kinematic and kinetic constraints derived from the literature, power consumption considerations, safety aspects, and motion requirements. The proposed AAFO features a two-stage transmission system comprising a planetary and a worm gear. Using a worm gear enables redistribution of the actuation mechanism along the tibial link, allowing even distribution of volume, enhancing compactness, and reducing distal mass. Additionally, the non-backdrivable nature of the mechanism enhances energy efficiency by allowing the joint to sustain static loads without active power consumption. The AAFO was integrated into the TWIN control architecture through an optimized trajectory planning method. This method leverages the desired hip and knee joint angles to generate adaptive ankle trajectories, ensuring continuous ground contact during the support phase and minimizing stumbling risk during the swing phase. A position bandwidth test was conducted to assess the device’s capability to track human-like trajectories accurately. Furthermore, experimental validation was performed with healthy users to evaluate the AAFO’s effectiveness in real-world scenarios. The results demonstrated the system’s ability to respond to the design requirements, accommodating users with varying anthropometric characteristics and body weights while maintaining precise position control and exhibiting low power consumption during walking and sit-to-stand/stand-to-sit transitions. Future work will focus on the clinical testing and validation of the proposed AAFO with SCI. These studies will aim to assess the effectiveness of integrating powered ankle joints into exoskeleton-assisted rehabilitation, evaluating their impact on gait performance, user comfort, and overall therapeutic outcomes.

## Data Availability

The raw data supporting the conclusions of this article will be made available by the authors, without undue reservation.
